# Photodynamic therapy in the therapy for recurrent/persistent nasopharyngeal cancer

**DOI:** 10.1186/1758-3284-1-40

**Published:** 2009-12-17

**Authors:** Maarten AM Wildeman, Heike J Nyst, Baris Karakullukcu, Bing I Tan

**Affiliations:** 1 Department of Head and Neck Oncology and Surgery, The Netherlands Cancer Institute - Antoni van Leeuwenhoek Hospital, Plesmanlaan 121, 1066 CX Amsterdam, the Netherlands

## Abstract

To determine the efficacy of Photodynamic therapy of patients with recurrent Nasopharyngeal Carcinoma we reviewed all available literature.

Since the treatment options for recurrent or persistent Nasopharyngeal Carcinoma are limited, the survival rates poor and the complications severe; there is definitely a place for alternative treatment modalities with more efficacy and less morbidity. Photodynamic therapy (PDT) has the potential to be a very effective local treatment modality for recurrent or persistent nasopharyngeal cancer, without the severe side effects seen with re-irradiation. This review shows all reported results of Photodynamic therapy in the treatment for Nasopharyngeal Carcinoma.

## Introduction

Nasopharyngeal carcinoma (NPC) occurs sporadically in the Western world, but is endemic in certain parts of South-East Asia, such as southern China and the Indonesian archipelago [1]. The worldwide incidence of NPC exceeds 80.000 cases/year with 19,616 new cases each year in South-China [2], but for most developing countries data are lacking due to absent or inadequate registries.

Treatment for primary NPC is radiotherapy, with chemotherapy for advanced stage disease. Despite the radiotherapy responsiveness of nasopharyngeal tumours, good long term survival is only achieved for patients who have early primary tumours with minimal neck disease with 67-71% 10 years disease free survival for T1, T2 and N0-1. Survival is poor for patients who have extended tumours and/or extended neck nodes 29-54% 10 years disease free survival for T3, T4 and N2, N3) [3-6].

Poor survival in the T4N0-1 category is chiefly the result of the high local recurrence rate (63.8%), where as for the T1-2N2-3 category, it is the result of the high distant metastases rate (approximately 50%). For the local recurrent or persistent tumours there are few treatment options left all with severe side effects.

## Re-treatment of recurrent/persistent NPC

Usual treatment options for early stage recurrent or persistent NPC are surgery in combination with external radiotherapy, brachytherapy alone [7-9], or in combination with external re-irradiation [10-13] or stereotactic radiosurgery [14]. The standard treatment for advanced stages of recurrent or persistent disease is chemotherapy followed by re-irradiation, or concomitant chemo-re-irradiation [15]. Pryzant reported on 53 patients with locally persistent or recurrent NPC treated with re-irradiation. Forty-two patients were re-treated with external beam therapy alone and 11 with a component of brachytherapy. Local recurrence was confined to the nasopharynx in 27 patients, and persistent tumour in 26 patients. The five-year actuarial local tumour control rate was 35%, five-year disease free survival was 18%, and overall survival was 21%. Eight Patients developed severe complications from retreatment, two involving the brain, one the spinal cord, and two the cranial nerves, all of which were fatal. The five-year actuarial incidence of severe complications was 17%. The incidence of severe complications was related to the total cumulative dose of external irradiation [16].

Lee described the incidence of late complications after re-irradiation in 891 patients with local recurrence after definitive radiation therapy for nasopharyngeal carcinoma. After external re-irradiation, brachytherapy or a combination of both, a wide variety of serious complications, such as temporal lobe necrosis, cranial neuropathy, endocrine dysfunction, trismus and bone/soft tissue necrosis was reported, with an overall incidence of 23 to 29% and a treatment mortality from 1 to 3% [17]. Other authors also report these serious side effects [10-12]. A major determinant of post-treatment complication is the severity of damage sustained during the initial course. In case of re-irradiation after an interval of 2 years or more, the sum of total doses tolerated is higher than predicted based on results of single course irradiations [18].

Since the treatment options for recurrent or persistent NPC are limited, the survival rates poor and the complications severe; there is definitely a place for alternative treatment modalities with more efficacy and less morbidity.

## Photodynamic Therapy

Photodynamic therapy (PDT) is a non-invasive treatment modality that might have a substantial role in treatment of recurrent nasopharyngeal carcinoma. PDT facilitates tumour destruction by the combination of a photosensitizer and laser light of a specific wavelength.

The therapeutic response of PDT depends on a complex combination of parameters that includes drug dose, drug-light interval, tissue oxygenation, light dose and light intensity (the last two are more accurately referred to as fluence and fluence rate, respectively). PDT works through non thermal chemical pathways to damage cancer cells. The main agents of cell destruction are activated singlet oxygens. The cancer cells are destroyed by a process that might take up to several weeks and the treated area heals with normal mucosa advancing from adjacent tissue much like radiation treatment.

## Photodynamic Therapy for Recurrent and Persistent NPC

Sun [19] has published the largest case series with 137 patients. Unfortunately this article is in Chinese. He has treated 137 NPC patients with hematoporphyrin derivative mediated PDT. Forty-eight and 72 hours after iv administration of 3-5 mg/kg HpD, laser treatment with either argon or dye laser was carried out. Dye laser (630 nm) with over 350 mw output transmitted through quartz fiber was given to 57 patients. Argon laser (488 nm and 514.5 nm) was delivered to 80 patients. The results were: complete response 76 cases (55.47%) and marked response 47 cases (34.31%), with an over-all marked response rate of 89.78% (123/137). These results are very successful. However, it is not clear if the patients had recurrent NPC or PDT is delivered as primary first choice treatment. In either case the results are very encouraging with such a large case series.

Kulapaditharom [20]et al reported in 1996 five patients treated with PDT for recurrent or residual NPC in Ramathidoni hospital in Bangkok. Three of these patients had a T1 tumour with no lymph node involvement and no distant metastasis. Two of these patients had a T3 tumour with no lymph node involvement and no distant metastases. 48 hours before illumination patients received Hematopirfyrin derivate with a dosage of 3 mg/kg and were illuminated (630 nm) by an optical fiber. The dose varied within 50-100 J/cm2. Power of the laser beam was adjusted to give a density within 100-150 mW/cm2 to avoid thermal effect.

The T1 tumour patients had a complete response after one treatment. Complete response has been suggested by endoscopic examination and negative biopsy. The duration of response were: 24, 16 and 11 months. The T3 tumours responded partially. One patient received 4 treatments and the other one 2 treatments before they switched to chemotherapy. The partial response duration was respectively 19 and 12 months.

The side effects described for all treatment were oedema, pain and photosensitivity. All side effects would solve without treatment. There was one patient with severe pain after PDT. All other side effects were mild and moderate.

The same Kulapaditharom [21] et al reported in 2000 the treatment of 12 NPC patients. The patients received Hematopirfyrin derivate with a dosage of 3 mg/kg 48 hours before they were illuminated (630 nm) by an flat cut optical fiber (with a core diameter of 400 μm, Laserscope California) with a with a dose of 100 J/cm2 and a fluence rate of 100 mW/cm2.

One patient was primary treated for an unknown primary. The primary tumour revealed in the nasopharynx (T1 NO MO) and because this patient already received radiotherapy and neck dissection he was treated with PDT. Complete response as observed for a follow up period of 31 months.

Six patients were treated for recurrent or residual T1-T2 NPC without lymph nodes and distant metastasis. These patients had received chemo-radiation for initial treatment. All patients had a biopsy and endoscopic complete respons after PDT treatment. Duration respons was between 10 and 66 months, mean duration was 26,5 months. One patient died 12 months after PDT cause of an unrelated cause.

One patient had a residual T3N0M0 adenoid cystic carcinoma instead of squameus cell carcinoma. Patient was treated with PDT and surgery. Patient had a partial response and has been symptom free for 22 months.

Four patients received PDT as adjunct to conventional treatment; these patients were all T3-T4 patients. One received radiotherapy, the other three received 6 cycles of chemotherapy. Three patients had a partial response and one had a complete response.

Tong [22]et al reported 12 patients treated with PDT for recurrent NPC in Prince of Wales Hospital in Hong Kong. These patients were treated with hematoporphyrin derivate 5 mg/kg and exposed with 200 J/cm2 delivered from a gold laser vaper with a wave length of 630 nm.

In eight patients cure of disease was deemed possible. The other four patients were treated for palliation of nasal obstruction. Four patients received surgical debulking before PDT. All patients showed radiology confirmed tumour regression six months after treatment. From the eight patients treated for cure five patients had a residual disease after 3-5 months, the other three remained disease free with a follow up between 9-12 months. Four patients had nasal regurgitation and two patients had their trismus aggravated, these side effects were temporarily. Two patients had mild skin hypersensitivity.

**Lofgren **[23]**et al **reported 5 patients treated with PDT for recurrent or persistent NPC in Orebro Medical Center Hospital, Sweden. Four patients were treated with hematoporphyrin derivative 2.5 mg/kg. One patient has been treated with potfimer sodium 2.0 mg/kg. The light dose varied from 50 to 100 J/cm2 and radiance varied from 100 to 150 mW/cm2.

Four patients had SCC, two of them had a recurrence and two of them persistent disease. One patient had a persistent adenocarcinoma. All patients were initially treated with radiotherapy.

Three patients remain tumour free with a follow up between 51 and 60 months. These patients had a tumour depth of 10 mm or less. One patient had persistent disease after PDT, a MRI-scan showed after treatment a tumour depth of 13 mm. One patient had a recurrence 6 months after treatment.

All patients had significant headache (3 to 8 months) and middle ear effusion. Two patients experienced minor problems of photosensitivity after sun exposure five 40 days after treatment (Additional file 1).

## Discussion

PDT has the potential to be a very effective local treatment modality for NPC, without the severe side effects seen with radiotherapy.

The articles above have shown that PDT is effective in destroying NPC, with good local control of tumor growth and complete responses in the majority of small recurrent or persistent disease (T1, T2) and long-term palliation in advanced stage (T3, T4) of the recurrence.

There are several advantages of PDT for management of recurrent NPC. Perhaps the most important advantage is its repeatability. PDT does not have cumulative effects. In case of a partial response the same area can be illuminated again. Secondly PDT is a local treatment rather than a systemic one. The photosensitizing drug accumulates in higher concentrations within cancer cells rendering them more susceptible to the toxic effects of light, compared to normal healthy cells and the light is delivered directly onto the tumour sparing the surrounding normal mucosa. Two other advantages of PDT are normal healing of the wound and illumination is only once.

In the papers reviewed above they all used the first generation photosensitizer hematoporphyrin. Yow [24] et al has shown in nasopharyngeal cancer cell lines that the uptake of second generation potosensitizer temaporfin is higher and efficiency is better compared with hematoporphyrin. Yow [25]et al also compared temoporfin and merocyanine 540, both second generation photosentisizers. They concluded that temoporfin-mediated PDT has a more potent effect in comparison with merocyanine- 540-mediated PDT. Based on these studies we hope for even better treatment results if temoporfin is used.

**Figure 1:**
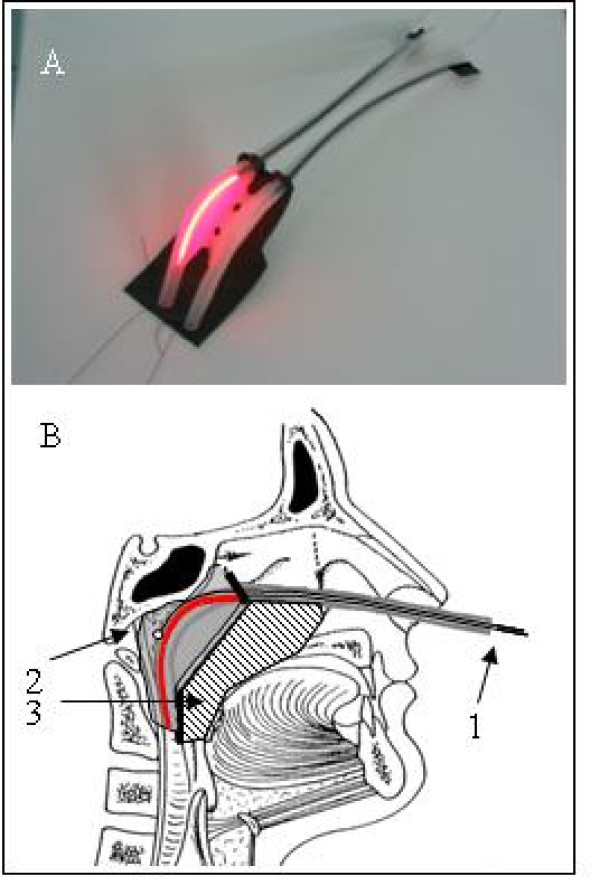
**A: Nasopharynx applicator, B: schematic view of positioning and illumination**. 1. Cylindrical diffuser in shielding tube. 2. Target area. 3. Soft palate is shielded. Figure 1 have beenused previously by Nyst et al, 2007 [26]

**Figure 2:**
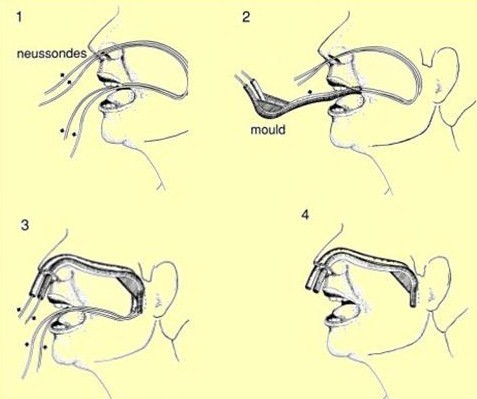
**Insertion of the Nasopharyngeal Applicator**.

**Figure 3:**
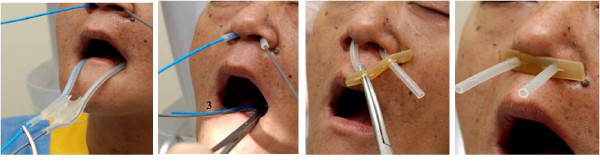
**Volunteer with Nasopharyngeal Applicator**.

Illumination of nasopharyngeal cavity is a challenge, since access is difficult and the cavity has a complex and irregular geometry. Furthermore the cavity varies significantly in size and geometry between patients. The complex shape of the nasopharyngeal cavity makes it impossible to produce a homogenous field of illumination. In order to deliver a sufficient light distribution throughout the nasopharyngeal cavity over exposure cannot be avoided. Over exposure of tumour or surrounding normal mucosa is however not considered a problem. Some critical structures are well protected by bone others; however, like the soft palate and the dorsal oropharyngeal wall would suffer unacceptable damage by this approach. Hence, these areas must be shielded against the laser light. For this purpose we have developed a novel dedicated light delivery applicator that ensures proper light delivery to the target area and enables for proper shielding of the risk areas [26,27]. A new study has been commenced to use this applicator to deliver PDT to patients with recurrent NPC cancers by our group. How we illuminate the Nasopharynx is shown by Figure 1, 2, 3.

The side effects are much less frequent and severe compared to chemotherapy. Sunlight exposure have shown that photosensitivity is not a real burden for these patients and only limited adverse events were related to this problem [28]. In general, patients in this part of the world have a colored skin that makes them less prone to sunburn. The fact that people in the tropics generally avoid sun exposure also helps to minimize the problems around the photosensitivity.

The immunologic reaction followed by PDT could be partly responsible for the good results. The theory is that an immunological response will be provoked by the use of PDT [29]. So in addition to these clinical trials several groups are working on the effectiveness of PDT in laboratory settings. PDT and the use of natural compounds become one of the new approaches in the investigation of NPC treatment. Such a group from Singapore has reported very encouraging results with hyperecin mediated PDT. This group has published extensively on biochemical pathways of cell damage caused by hypericin-PDT on NPC cell lines in vitro. Their excellent review titled " Hypericin lights up the way for the potential treatment of nasopharyngeal cancer by photodynamic therapy" by Olivo [30] et al summarizes this effort and proposes that hypericin-PDT is a potential treatment of NPC in humans. Koon [31] et al has chosen a different photosensitizing agent from the traditional Chinese medicine: curcumin. Their in vitro study with CNE2 NPC cells shows it to be a promising agent.

Xu et al[32] preferred to worked with photodynamic effects of pyropheophorbide-a methyl ester (MPPa) in CNE2 NPC cells, demonstrating the apoptotic effect and suggesting its clinical potential. Mak et al[33] worked with the same cell line inducing phototoxicity with sulfonamide derivatives of porphycene with successful results. Betz [34] et al obtained good results in their in vitro study with amore conventional photosensitizer: 5-aminolevulinic acid.

Bae [35] et al examined the immunotherapeutic significance of HPV-immortalitized tumour cell lysates induced by PDT and CpG-oligodeoxynucleotide(ODN). They found a significant induced IFN-β production and cytotoxic T- cell response in the PDT-cell lysate plus ODN immunized groups. Although PDT in combination with immunotherapy is still in experimental fase it offers a great promise as a new alternative treatment. On to this time no research has been done on PDT in combination with Immunotherapy in EBV related NPC. This combination could be very promising not only for local recurrence but also for advanced disease.

## Competing interests

The authors declare that they have no competing interests.

## Authors' contributions

MW did the literature search and wrote the article, HN and BK both repeated literature search and helped writing the introduction and discussion. IT designed overall article. All authors read and approved the final manuscript.

## Author information

Prof. Tan is also connected to Amsterdam Medical Center, as such his alternative email address is i.b.tan@amc.uva.nl

## Supplementary Material

Additional file 1 Overall Treatment Results Click here for file
