# Supplementary material for: Photodynamic therapy in the therapy for recurrent/persistent nasopharyngeal cancer
Source: Head Neck Oncol. 2009 Dec 17;1:40. doi: 10.1186/1758-3284-1-40 (PMC2809049; doi:10.1186/1758-3284-1-40)
Supplement: Additional file 1 — Overall Treatment Results [file 1758-3284-1-40-S1.DOC]

**Additional file 1: Overall Treatment Results**
